# Case Report: Exceptional survival with immunotherapy in a patient with *EGFR* exon 20 insertion (p.S768_D770dup) and high PD-L1 expression

**DOI:** 10.3389/fimmu.2026.1892764

**Published:** 2026-07-22

**Authors:** Shasha Zhao, Yuanhang Chen, Meiying Kuang, Yi Li

**Affiliations:** Department of Oncology, Xingyi People’s Hospital, Xingyi, China

**Keywords:** *EGFR* ex20ins, long-term survival, non-small cell lung cancer, PD-L1 high expression, tislelizumab

## Abstract

*EGFR* exon 20 insertion (ex20ins) NSCLC typically shows limited response to immunotherapy. We report a rare case of a smoker with metastatic *EGFR* ex20ins (p.S768_D770dup) and high PD-L1 expression (TPS 50%) who achieved exceptional survival (OS = 63 months). Following progression on chemotherapy and intolerance to afatinib, the patient received paclitaxel plus tislelizumab. This combination yielded durable disease control. While the overall benefit of immune checkpoint inhibitors (ICIs) in EGFR-mutant NSCLC remains limited, this case suggests that chemo-immunotherapy (particularly taxane + PD-1 inhibition) may provide significant benefit in ex20ins tumors with high PD-L1 expression and smoking history. These findings highlight the critical value of targeted molecular profiling and offer a viable precision oncology strategy when novel targeted agents are inaccessible.

## 1 Introduction

Non-small cell lung cancer (NSCLC) remains a leading cause of cancer-related mortality worldwide. Activating epidermal growth factor receptor (*EGFR*) alterations are actionable drivers in NSCLC (1), however, NSCLC harboring *EGFR* ex20ins mutations has historically shown limited sensitivity to first- and second-generation *EGFR* tyrosine kinase inhibitors (TKIs) (2). Recently, new targeted agents and antibody-based approaches have expanded therapeutic options (3), yet real-world access and optimal sequencing strategies continue to evolve.

In the immunotherapy era, immune checkpoint inhibitors (ICIs) have revolutionized clinical outcomes for many patients with advanced NSCLC. However, clinical responses to ICIs in *EGFR*-altered tumors are generally less frequent and durable compared with those in *EGFR*–wild-type disease (4, 5). Importantly, EGFR-mutant NSCLC is a highly heterogeneous disease, and atypical mutations such as ex20ins should not be considered entirely equivalent to classical mutations (19del/L858R) in the context of immunotherapy. While classical EGFR mutations are typically associated with an uninflamed tumor microenvironment, emerging evidence indicates that ex20ins tumors may harbor distinct immunological features that potentially render them more responsive to ICIs.

Here, we present a case of a patient with metastatic *EGFR* ex20ins NSCLC who achieved an exceptionally prolonged survival (OS = 63 months) following multimodal and sequential systemic therapies. This case underscores the clinical value of subtype-level *EGFR* annotation, longitudinal reassessment, and individualized treatment sequencing.

## 2 Case presentation

**Figure 1:**
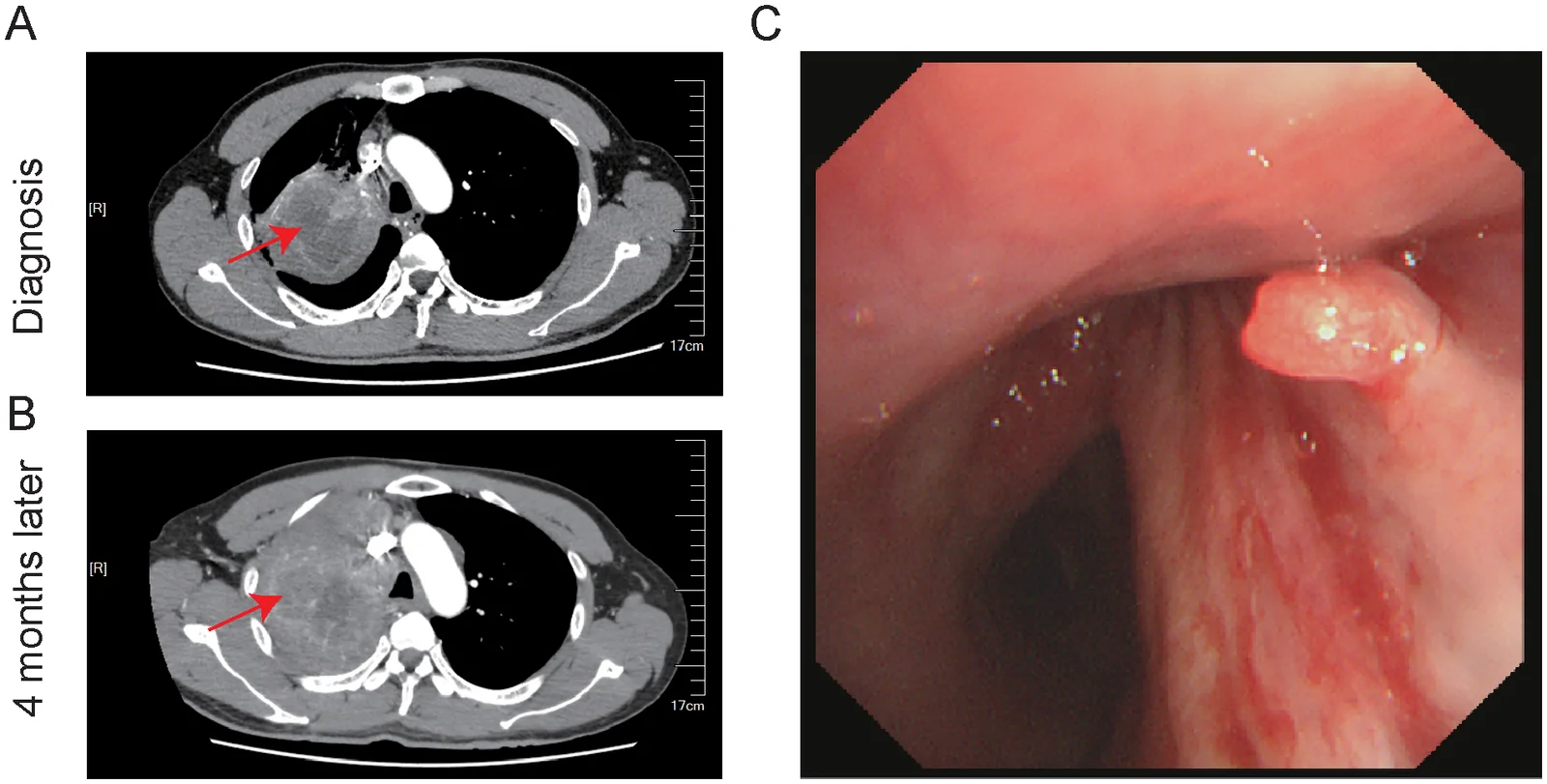
Baseline radiological and endoscopic findings. **(A)** Surveillance contrast-enhanced CT revealed a new 8.8*7.5 cm nodule in the right upper lobe (March 11, 2021). **(B)** Restaging contrast-enhanced CT performed 4 months later showed progression of the pulmonary and lymph node metastases. CT, computed tomography (July 04, 2021). **(C)** Fiberoptic bronchoscopy revealed a neoplasm obstructing the opening of the right upper lobe bronchus, from which a biopsy specimen was obtained (March 15, 2021).

A 48-year-old male with a 20 pack-years smoking history was diagnosed with stage IVB lung adenocarcinoma arising from the right upper lobe, harboring an *EGFR* ex20ins mutation and high PD-L1 expression. Following disease progression on chemotherapy and intolerance to afatinib, the patient achieved durable disease control (OS = 63 months) with tislelizumab (third-line paclitaxel plus tislelizumab). The patient presented with cough and sputum symptoms on March 26, 2021. A chest computed tomography (CT) scan revealed an 8.8*7.5*8.0cm mass in the right upper lobe (**Figure 1A**) with obstructive atelectasis, accompanied by multiple metastases in the right hilar, mediastinal, and right supraclavicular lymph nodes. A nodule in the dorsal segment of the right lower lobe (1.0*0.9*0.65cm) was identified as a metastasis. Magnetic resonance imaging (MRI) of the brain showed multiple metastatic nodules in the left cerebellar hemisphere and bilateral cerebral hemispheres. A whole-body bone scan indicated metastases involving multiple ribs (left 5th, 9th, 10th; right 2nd) and the left 1st costal cartilage.

**Figure 2:**
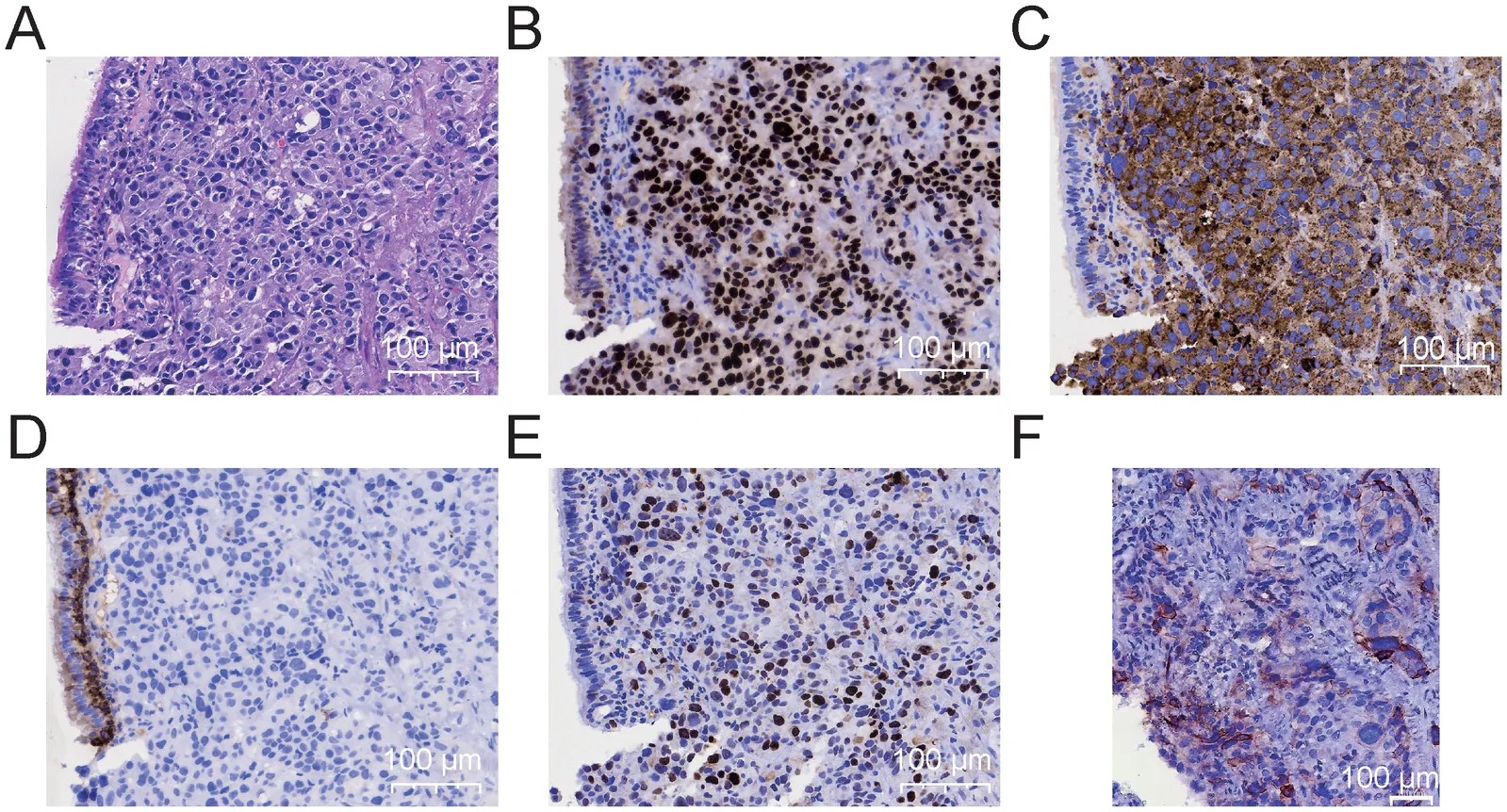
Histopathological and immunohistochemical characterization. **(A)** Hematoxylin and eosin (H&E) staining showing characteristic features of lung adenocarcinoma. **(B)** Immunohistochemical staining showing strong nuclear positivity for TTF-1. **(C)** Positive cytoplasmic staining for Napsin A. **(D)** Negative cytoplasmic staining for CK5/6. **(E)** Ki67 staining revealed that more than 20% of the cells showed a positive reaction. **(F)** High PD-L1 expression with a Tumor Proportion Score (TPS) of 50% (membranous staining). (Scale bars: 100 µm).

**Figure 3:**
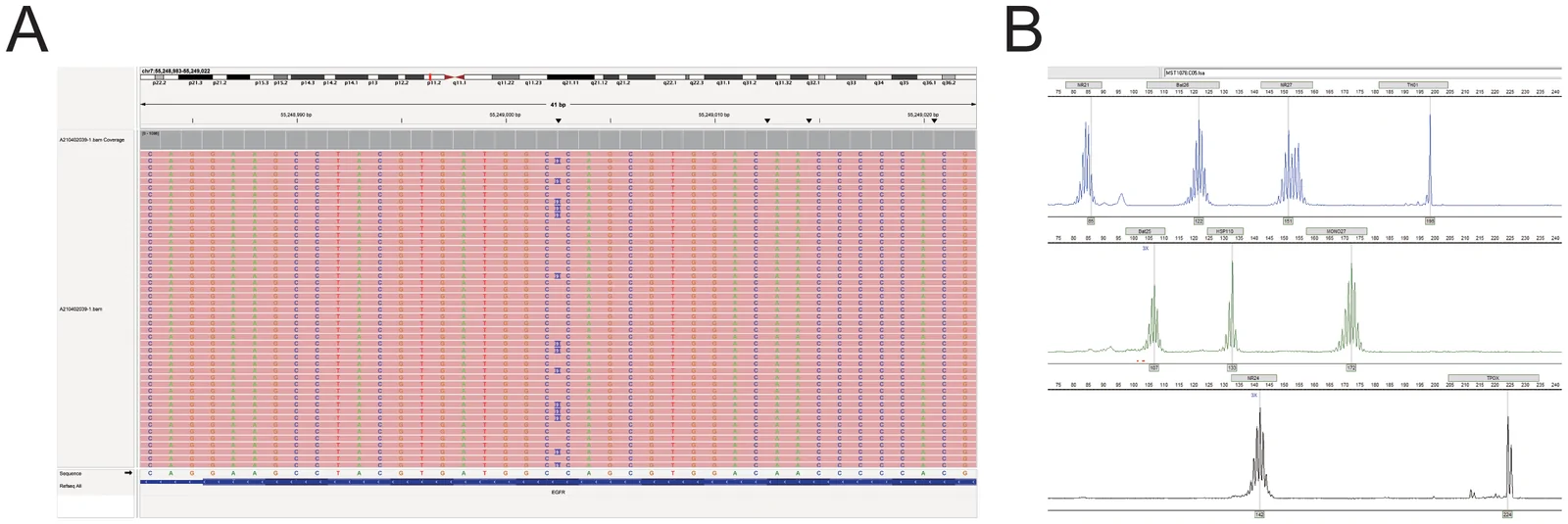
Molecular genetic analysis results. **(A)** Next-generation sequencing (NGS) read alignment (Integrative Genomics Viewer) displaying the *EGFR* ex20ins mutation (base change: c.2303_2311dup; amino acid change: p.S768_D770dup) with a variant allele frequency of 32.84%. **(B)** Microsatellite instability analysis showing stable peaks, confirming the tumor as Microsatellite Stable (MSS).

Tumor markers were elevated: CEA 9.19 ng/mL (normal, <5.0 ng/mL), CA125 92.19 U/mL (normal, <24 U/mL), CYFRA21-1 21.82 ng/mL (normal, <3.3 ng/mL), and NSE 17.84 ng/mL (normal, <16.3 ng/mL). Bronchoscopic biopsy of the right upper lobe mass confirmed lung adenocarcinoma (**Figures 1C**, **2A**). Immunohistochemistry showed positivity for TTF-1 and Napsin A (**Figures 2B**, **2C**), and negativity for CK5/6 (**Figure 2D**), P40, P63, and SYN. The Ki-67 proliferation index was >20% (**Figure 2E**). Notably, PD-L1 expression was high, with a tumor proportion score (TPS) of 50% (**Figure 2F**). Next-generation sequencing identified an *EGFR* ex20ins mutation (c.2303_2311dup, p.S768_D770dup) with a variant allele frequency of 32.84% (**Figure 3A**). The tumor was microsatellite stable (MSS) (**Figure 3B**). The patient was diagnosed with stage IVB lung adenocarcinoma (cT4N3M1c).

Treatment was delayed until July 08, 2021 due to personal reasons, at which point the patient presented with consciousness disturbance. Re-staging imaging showed significant progression of the primary tumor (11.5*7.9*10.0cm) (**Figure 1B**), increased mediastinal lymphadenopathy, and progression of brain metastases accompanied by peritumoral edema.

**Figure 4:**
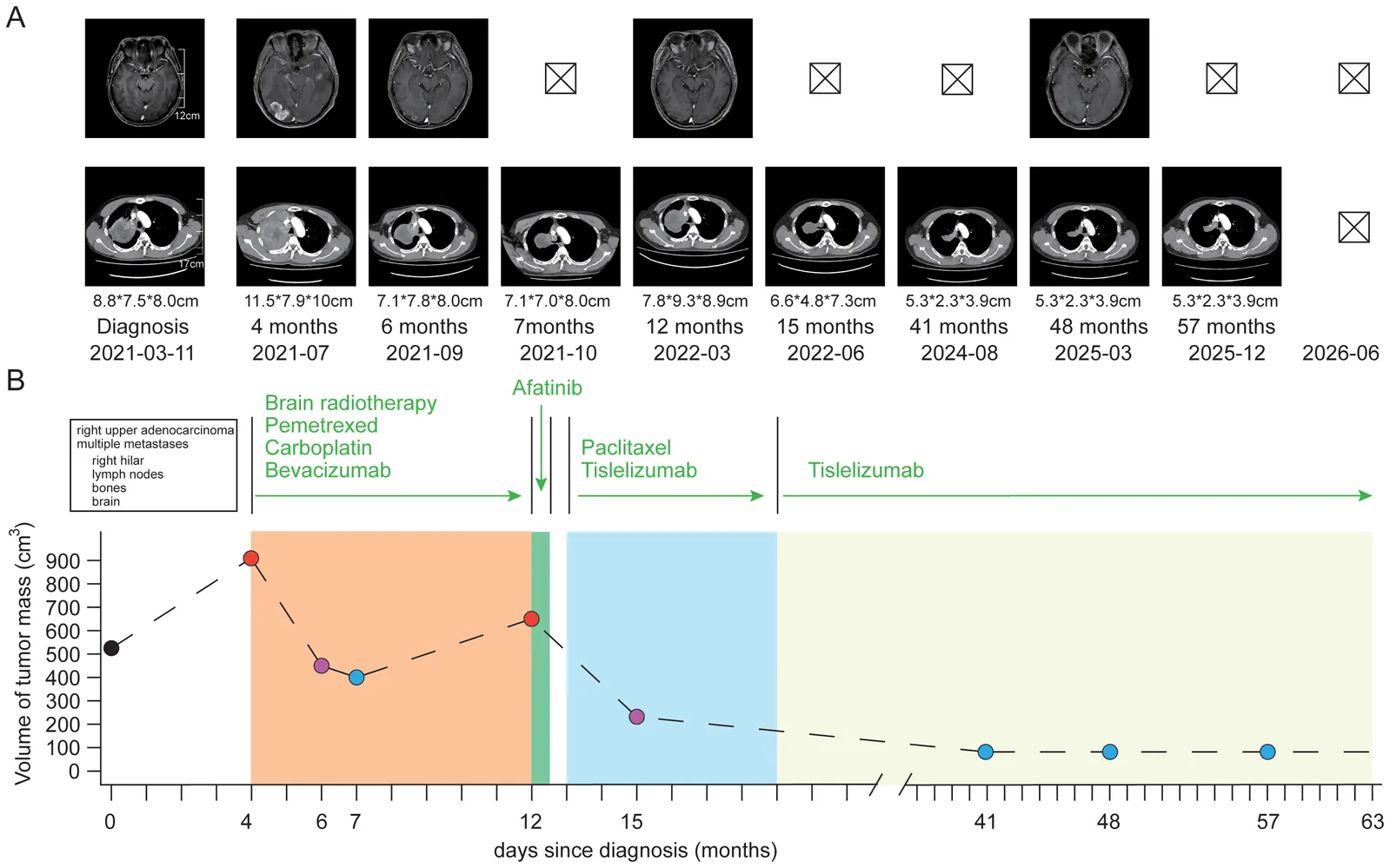
Clinical timeline and treatment response. **(A)** Serial radiological assessment. Top row: Brain MRI showing the evolution of metastatic lesions. Bottom row: Chest CT scans showing the primary tumor size changes from diagnosis (March 26, 2021) through progression (July 08, 2021) and subsequent response to treatments up to June 26, 2026. **(B)** Swimmer plot illustrating the patient’s clinical course, therapeutic regimens, and duration of response. The patient achieved durable disease control (OS = 63 months) with third-line paclitaxel plus tislelizumab after progression on chemotherapy and intolerance to afatinib. ☒: Not available; RECIST 1.1: PR (red), CR (pink), SD (blue).

The patient commenced first-line treatment with pemetrexed (500 mg/m²), carboplatin (AUC 5), and bevacizumab (7.5 mg/kg), concurrent with brain radiotherapy. A partial response (PR) was achieved by September 02, 2021, according to RECIST 1.1, followed by stable disease (SD) in October 19, 2021. The progression-free survival (PFS) for first-line therapy was 9 months. On March 23, 2022, although brain MRI showed a significant reduction in intracranial lesions, systemic disease progression was observed. Second-line treatment with the pan-HER inhibitor afatinib was initiated but discontinued after two weeks due to grade 3 gastrointestinal toxicity. Consequently, the therapeutic efficacy of afatinib could not be adequately assessed. Subsequently, on April 25, 2022, the patient began third-line therapy with paclitaxel plus the PD-1 inhibitor tislelizumab for 6 cycles. From October 2022 onward, he continued with tislelizumab maintenance therapy every 3 weeks. The patient has maintained stable disease on regular follow-up, achieving an overall survival (OS) of 63 months as of June 26, 2026 (**Figure 4**).

*EGFR* ex20ins mutations account for approximately 4-10% of all *EGFR* mutations and are historically associated with a poorer prognosis compared to classical *EGFR* mutations (19del/L858R) due to intrinsic resistance to early-generation tyrosine kinase inhibitors (TKIs). While novel agents like amivantamab and sunvozertinib have improved outcomes, access varies globally (6, 7).

This case is notable for the patient’s exceptional response to immune checkpoint inhibitor (ICI) therapy, a modality generally considered less effective in *EGFR*-mutant NSCLC. However, emerging evidence suggests that *EGFR* ex20ins tumors may harbor a distinct immune microenvironment compared with classical *EGFR* mutations. This patient possessed two favorable factors for immunotherapy: a history of heavy smoking and high PD-L1 expression (TPS 50%). While high PD-L1 expression is a well-established biomarker for ICI benefit in wild-type NSCLC, its predictive value in *EGFR*-mutant tumors remains highly controversial. Nevertheless, in this specific case, the high PD-L1 expression coupled with smoking-associated genomic signatures—which often correlate with a higher tumor mutational burden (TMB)—may have collectively enhanced tumor immunogenicity.

The patient’s specific mutation, p.S768_D770dup, is a duplication within the loop following the C-helix. While afatinib has shown limited activity in certain non-classical mutations, toxicity limited its use here. The switch to a chemo-immunotherapy combination (paclitaxel + tislelizumab) proved decisive. The synergy between chemotherapy (which can release tumor antigens) and PD-1 blockade likely contributed to the durable disease control observed. In contrast to the limited benefit from prior chemotherapy alone, the paclitaxel + tislelizumab combination yielded durable disease control.

## 3 Discussion

*EGFR* ex20ins mutations are associated with a poor prognosis due to intrinsic resistance to early-generation tyrosine kinase inhibitors (TKIs). The therapeutic landscape has recently been transformed by novel targeted agents. Currently, novel targeted agents, such as amivantamab (often combined with chemotherapy) and sunvozertinib, have demonstrated robust efficacy and are preferred as the standard of care for EGFR ex20ins NSCLC where available (6, 8). However, the high cost and variable global accessibility of these agents pose significant real-world challenges. Consistent with current literature, the overall benefit of immune checkpoint inhibitors (ICIs) in EGFR-mutant NSCLC is generally limited. Nevertheless, this case illustrates an exceptional survival (OS = 63 months) achieved *via* chemotherapy plus PD-1 blockade (tislelizumab). Rather than challenging the existing treatment paradigm, it suggests that chemo-immunotherapy remains a vital, cost-effective strategy when novel targeted options are unavailable or contraindicated, highlighting a specific subset of patients who may still derive durable benefit. Importantly, this durable response was achieved with a chemo-immunotherapy combination, not single-agent IO, aligning with evidence that single-agent checkpoint inhibitors have minimal activity in EGFR-mutant NSCLC.

Recent landmark trials have explored the role of chemo-immunotherapy in EGFR-mutant NSCLC following TKI progression. For instance, the IMpower150 trial demonstrated clinical benefit with the addition of atezolizumab to bevacizumab and chemotherapy in EGFR-mutant patients (9). Conversely, the CheckMate 722 trial (nivolumab plus chemotherapy) did not show a statistically significant improvement in overall survival compared to chemotherapy alone in post-TKI EGFR-mutant NSCLC, underscoring the heterogeneity of immune responses in this population (10). More specifically, a phase II study demonstrated the promising efficacy and acceptable safety of tislelizumab combined with chemotherapy in EGFR-mutant NSCLC after TKI failure (11). Furthermore, a series highlighted the potential clinical benefit of chemo-immunotherapy specifically in patients with EGFR mutations (12–15). Our case adds to this evolving landscape by suggesting that specific immunogenic features—such as the ex20ins variant combined with high PD-L1 expression and a smoking history—may define a unique subset of patients who can achieve exceptional, durable responses to chemo-IO regimens.

The durable response observed here underscores the biological heterogeneity between ex20ins tumors and classical *EGFR* mutations (19del/L858R). Unlike the generally immunosuppressive (“cold”) microenvironment of classical mutants, ex20ins tumors may harbor distinct immunogenic features. Recent retrospective analyses and immune profiling studies suggest that ex20ins NSCLC frequently exhibits a more favorable immune contexture, including higher rates of concurrent PD-L1 expression and relatively increased CD8+ T-cell infiltration compared to classical mutations (16). In this patient, two decisive factors were hypothesized to potentiated the efficacy of tislelizumab: a heavy smoking history and high PD-L1 expression (TPS 50%). It is important to note that the predictive value of PD-L1 expression in EGFR-mutant NSCLC remains controversial, and a direct causal relationship between PD-L1 positivity and ICI efficacy cannot be definitively established from a single case. However, smoking is a well-established surrogate for higher tumor mutational burden (TMB), which correlates with improved ICI response even in oncogene-driven NSCLC. Furthermore, the combination of taxane-based chemotherapy with PD-1 blockade likely induced immunogenic cell death, releasing tumor antigens that primed the immune system.

Structurally, the specific variant identified, p.S768_D770dup, is located within the loop region following the C-helix and is classically categorized as a “far-loop” insertion based on structure-based classifications (12). Structural modeling indicates that such far-loop insertions push the C-helix inward, locking the kinase in a constitutively active conformation. Crucially, this alteration creates a restricted drug-binding pocket and severe steric hindrance that prevents the optimal binding of early-generation TKIs (3, 12). While this structural mechanism theoretically predicts limited efficacy for afatinib, it must be interpreted with caution in our clinical case. The rapid failure of second-line afatinib in our patient was primarily driven by early discontinuation due to severe grade 3 gastrointestinal toxicity, precluding an adequate assessment of clinical response. Conversely, third-generation agents like furmonertinib are designed to accommodate these structural alterations. Sunvozertinib, a selective EGFR inhibitor, has demonstrated breakthrough efficacy in previously treated ex20ins NSCLC across various insertion locations, including loop insertions. Similarly, newer-generation agents like furmonertinib are designed to accommodate these structural alterations and have shown promising preclinical and clinical activity. However, our findings suggest that for Loop insertion variants, therapeutic decision-making should not be solely mutation-centric. The presence of the p.S768_D770dup variant did not hinder the efficacy of the ICI regimen, implying that while the mutation structure dictates TKI sensitivity, the immune microenvironment dictates ICI efficacy. Consequently, optimal sequencing for ex20ins should integrate molecular subtyping (Loop vs. Helical), immune profile (PD-L1/TMB), and economic feasibility. For selected patients with high PD-L1 expression and smoking history, Chemo-IO represents a potential, accessible alternative strategy when standard targeted therapies are inaccessible.

To contextualize our findings, it is instructive to compare this case with previously reported data regarding ICI efficacy in EGFR ex20ins NSCLC. Recent retrospective analyses have demonstrated that patients with EGFR ex20ins mutations exhibit distinct responses to ICIs compared to those with classical mutations. For instance, studies by Negrao et al. and Metro et al. reported that while ex20ins tumors have a comparatively higher objective response rate to ICIs than classical EGFR mutants, the median progression-free survival (PFS) in real-world cohorts remains modest, typically ranging from 4.8 to 5.3 months (17, 18). In this context, the exceptionally durable response observed in our patient—maintaining disease control for over 44 months on chemo-immunotherapy—is exceedingly rare. This profound and prolonged benefit distinguishes our case from average clinical outcomes and suggests that the convergence of specific clinical features (heavy smoking history), biomarker status (high PD-L1 expression), and the distinct immunogenic profile of the p.S768_D770dup variant may synergistically drive a highly favorable response to ICIs.

Limitations. As a single-patient observation, causal inference is limited. Additional constraints include the lack of serial ctDNA monitoring, the absence of TMB data, and the lack of paired baseline tissue for dynamic immune profiling. Nevertheless, the exceptional course supports reporting to inform hypothesis generation and future cohort studies. Furthermore, while TMB data was unavailable, the remarkable clinical success observed underscores the potential efficacy of this specific chemo-immunotherapy combination in selected patients.

## 4 Conclusion

We report a case of *EGFR* ex20ins (p.S768_D770dup) metastatic lung adenocarcinoma with high PD-L1 expression achieving an OS of 63 months. Notably, this prolonged survival was achieved despite the presence of multiple distant metastases, particularly challenging brain metastases. This case supports the consideration of PD-1 inhibitors, particularly in combination with chemotherapy, as a viable therapeutic strategy for *EGFR* ex20ins patients who are smokers with high PD-L1 expression, especially when targeted therapies are unavailable or poorly tolerated.

## Data Availability

The original contributions presented in the study are included in the article/supplementary material. Further inquiries can be directed to the corresponding author.
