# Correction: Case Report: Exceptional survival with immunotherapy in a patient with *EGFR* exon 20 insertion (p.S768_D770dup) and high PD-L1 expression

**DOI:** 10.3389/fimmu.2026.1952941

**Published:** 2026-09-03

**Authors:** Shasha Zhao, Yuanhang Chen, Meiying Kuang, Yi Li

**Affiliations:** Department of Oncology, Xingyi People’s Hospital, Xingyi, China

**Keywords:** *EGFR* ex20ins, long-term survival, non-small cell lung cancer, PD-L1 high expression, tislelizumab

The reference for “1” was erroneously written as “Lee SH, Lu S, Hayashi H, Ahn MJ, Kim SW, Park K, et al. Lazertinib versus osimertinib in previously untreated EGFR-mutant advanced NSCLC: a randomized, double-blind, exploratory analysis from MARIPOSA. J Thorac Oncol. (2025) 20:1655– 68. doi: 10.1016/j.jtho.2025.06.030”. It should be “Lee SH, Lu S, Hayashi H, Felip E, Spira AI, Girard N, et al. Lazertinib versus osimertinib in previously untreated EGFR-mutant advanced NSCLC: a randomized, double-blind, exploratory analysis from MARIPOSA. *J Thorac Oncol*. (2025) 20:1655-1668. doi: 10.1016/j.jtho.2025.06.030”.

The reference for “2” was erroneously written as “Remon J, Hendriks LEL, Cardona AF, Besse B, Pluzanski A, Vansteenkiste J, et al. EGFR exon 20 insertions in advanced non-small cell lung cancer: a new history begins. Cancer Treat Rev. (2020) 90:102105. doi: 10.1016/j.ctrv.2020.102105”. It should be “Remon J, Hendriks LEL, Cardona AF, Besse B. EGFR exon 20 insertions in advanced non-small cell lung cancer: a new history begins. *Cancer Treat Rev*. (2020) 90:102105. doi: 10.1016/j.ctrv.2020.102105”.

The reference for “3” was erroneously written as “Zhao H, Beyett TS, Jiang J, Zhang T, Liu Y, Wang L, et al. Biochemical analysis of EGFR exon20 insertion variants insASV and insSVD and their inhibitor sensitivity. Proc Natl Acad Sci USA. (2024) 121:e2417144121. doi: 10.1073/pnas.2417144121”. It should be “Zhao H, Beyett TS, Jiang J, Rana JK, Schaeffner IK, Santana J, et al. Biochemical analysis of EGFR exon20 insertion variants insASV and insSVD and their inhibitor sensitivity. *Proc Natl Acad Sci U S A*. (2024) 121:e2417144121. doi: 10.1073/pnas.2417144121”.

The reference for “4” was erroneously written as “Meyer ML, Fitzgerald BG, Paz-Ares L, Cappuzzo F, Jänne PA, Peters S, et al. New promises and challenges in the treatment of advanced non-small-cell lung cancer. Lancet. (2024) 404:803–22. doi: 10.1016/s0140-6736(24)01029-8”. It should be “Meyer ML, Fitzgerald BG, Paz-Ares L, Cappuzzo F, Jänne PA, Peters S, et al. New promises and challenges in the treatment of advanced non-small-cell lung cancer. *Lancet*. (2024) 404:803-822. doi: 10.1016/s0140-6736(24)01029-8”.

The reference for “5” was erroneously written as “Yang Y, Zhang X, Gao Y, Liu J, Li M, Wang H, et al. Research progress in immunotherapy of NSCLC with EGFR-sensitive mutations. Oncol Res. (2022) 29:63– 74. doi: 10.3727/096504022x16462176651719”. It should be “Yang Y, Zhang X, Gao Y, Dong Y, Wang D, Huang Y, et al. Research progress in immunotherapy of NSCLC with EGFR-sensitive mutations. *Oncol Res*. (2022) 29:63-74. doi: 10.3727/096504022x16462176651719”.

The reference for “6” was erroneously written as “Wang K, Du R, Myall NJ, Horn L, Riely GJ, Spigel DR, et al. Real-world efficacy and safety of amivantamab for EGFR-mutant NSCLC. J Thorac Oncol. (2024) 19:500–6. doi: 10.1016/j.jtho.2023.11.020”. It should be “Wang K, Du R, Myall NJ, Lewis WE, Uy N, Hong L, et al. Real-world efficacy and safety of amivantamab for EGFR-mutant NSCLC. *J Thorac Oncol*. (2024) 19:500-506. doi: 10.1016/j.jtho.2023.11.020”.

The reference for “7” was erroneously written as “Wang M, Yang JC, Mitchell PL, Lu S, Zhou C, Han B, et al. Sunvozertinib, a selective EGFR inhibitor for previously treated non-small cell lung cancer with EGFR exon 20 insertion mutations. Cancer Discov. (2022) 12:1676–89. doi: 10.21037/tcr.2020.03.10”. It should be “Wang M, Yang JC, Mitchell PL, Fang J, Camidge DR, Nian W, et al. Sunvozertinib, a selective EGFR inhibitor for previously treated non-small cell lung cancer with EGFR exon 20 insertion mutations. *Cancer Discov*. (2022) 12:1676-1689. doi: 10.1158/2159-8290.cd-21-1615”.

The reference for “8” was erroneously written as “Han B, Zhou C, Wu L, Yang Y, Zhang L, Li Y, et al. Preclinical and preliminary clinical investigations of furmonertinib in NSCLC with EGFR exon 20 insertions (20ins). Ann Oncol. (2021) 32:S949–S1039. doi: 10.1016/j.annonc.2021.08.1815”. It should be “Han B, Zhou C, Wu L, Yu X, Li Q, Liu F, et al. Preclinical and preliminary clinical investigations of furmonertinib in NSCLC with EGFR exon 20 insertions (20ins). *Ann Oncol*. (2021) 32: s964. doi: 10.1016/j.annonc.2021.08.1815”.

The reference for “9” was erroneously written as “Nogami N, Barlesi F, Socinski MA, Mok T, Reck M, Atagi S, et al. IMpower150 final exploratory analyses for atezolizumab plus bevacizumab and chemotherapy in key NSCLC patient subgroups with EGFR mutations or metastases in the liver or brain. J Thorac Oncol. (2022) 17:309–23. doi: 10.1016/j.jtho.2021.09.014”. It should be “Nogami N, Barlesi F, Socinski MA, Reck M, Thomas CA, Cappuzzo F, et al. IMpower150 final exploratory analyses for atezolizumab plus bevacizumab and chemotherapy in key NSCLC patient subgroups with EGFR mutations or metastases in the liver or brain. *J Thorac Oncol*. (2022) 17:309-323. doi: 10.1016/j.jtho.2021.09.014”.

The reference for “10” was erroneously written as “Mok T, Nakagawa K, Park K, Ahn MJ, Kim SW, Cho BC, et al. Nivolumab plus chemotherapy in epidermal growth factor receptor-mutated metastatic non-small-cell lung cancer after disease progression on epidermal growth factor receptor tyrosine kinase inhibitors: final results of CheckMate 722. J Clin Oncol. (2024) 42:1252–64. doi: 10.1200/jco.23.01017”. It should be “Mok T, Nakagawa K, Park K, Ohe Y, Girard N, Kim HR, et al. Nivolumab plus chemotherapy in epidermal growth factor receptor-mutated metastatic non-small-cell lung cancer after disease progression on epidermal growth factor receptor tyrosine kinase inhibitors: final results of CheckMate 722. *J Clin Oncol*. (2024) 42:1252-1264. doi: 10.1200/jco.23.01017”.

The reference for “11” was erroneously written as “Zhong H, Zhang X, Tian P, Wang Y, Chen J, Liu S, et al. Tislelizumab plus chemotherapy for patients with EGFR-mutated non-squamous non-small cell lung cancer who progressed on EGFR tyrosine kinase inhibitor therapy. J Immunother Cancer. (2023) 11:e006887. doi: 10.1136/jitc-2023-006887. ref 11 in the revised manuscript.”. It should be “Zhong H, Zhang X, Tian P, Chu T, Guo Q, Yu X, et al. Tislelizumab plus chemotherapy for patients with EGFR-mutated non-squamous non-small cell lung cancer who progressed on EGFR tyrosine kinase inhibitor therapy. *J Immunother Cancer*. (2023) 11:e006887. doi: 10.1136/jitc-2023-006887”.

The reference for “12” was erroneously written as “Li Y, Jiang H, Qian F, Wang L, Zhao J, Sun Y, et al. Efficacy of ICI-based treatment in advanced NSCLC patients with PD-L1≥50% who developed EGFR-TKI resistance. Front Immunol. (2023) 14:1161718. doi: 10.3389/fimmu.2023.1161718”. It should be “Li Y, Jiang H, Qian F, Chen Y, Zhou W, Zhang Y, et al. Efficacy of ICI-based treatment in advanced NSCLC patients with PD-L1≥50% who developed EGFR-TKI resistance. *Front Immunol*. (2023) 14:1161718. doi: 10.3389/fimmu.2023.1161718”.

The reference for “13” was erroneously written as “Chen Y, Yang Z, Wang Y, Liu H, Zhang Q, Xu L, et al. Pembrolizumab plus chemotherapy or anlotinib vs. pembrolizumab alone in patients with previously treated EGFR-mutant NSCLC. Front Immunol. (2021) 11:671228. doi: 10.3389/fonc.2021.671228”. It should be “Chen Y, Yang Z, Wang Y, Hu M, Zhang B, Zhang Y, et al. Pembrolizumab plus chemotherapy or anlotinib vs. pembrolizumab alone in patients with previously treated EGFR-mutant NSCLC. *Front Oncol*. (2021) 11:671228. doi: 10.3389/fonc.2021.671228”.

The reference for “14” was erroneously written as “Zhang M, Huang Q, Yu M, Li X, Wang H, Zhao Y, et al. Immunotherapy for nonsmall cell lung cancer with EGFR or HER2 exon 20 insertion mutations: a real-world analysis. Transl Lung Cancer Res. (2023) 12:797–807. doi: 10.21037/tlcr-23-167”. It should be “Zhang M, Huang Q, Yu M, Xue J, Huang M, Lu Y, et al. Immunotherapy for non-small cell lung cancer with EGFR or HER2 exon 20 insertion mutations: a real-world analysis. *Transl Lung Cancer Res*. (2023) 12:797-807. doi: 10.21037/tlcr-23-167”.

The reference for “15” was erroneously written as “Kirchner M, Kluck K, Brandt R, Wohlleber M, Wolf J, Schuler M, et al. The immune microenvironment in EGFR- and ERBB2-mutated lung adenocarcinoma. ESMO Open. (2021) 6:100253. doi: 10.1016/j.esmoop.2021.100253”. It should be “Kirchner M, Kluck K, Brandt R, Volckmar AL, Penzel R, Kazdal D, et al. The immune microenvironment in EGFR- and ERBB2-mutated lung adenocarcinoma. *ESMO Open*. (2021) 6:100253. doi: 10.1016/j.esmoop.2021.100253”.

The reference for “16” was erroneously written as “Robichaux JP, Le X, Vijayan RSK, Hicks JK, Heeke S, Elamin YY, et al. Structurebased classification predicts mutant EGFR variant response to targeted therapies. Nature. (2021) 597:732–7. doi: 10.1038/s41586-021-03898-1”. It should be “Robichaux JP, Le X, Vijayan RSK, Hicks JK, Heeke S, Elamin YY, et al. Structure-based classification predicts drug response in EGFR-mutant NSCLC. *Nature*. (2021) 597:732-737. doi: 10.1038/s41586-021-03898-1”.

The reference for “17” was erroneously written as “Negrao MV, Reuben A, Robichaux JP, Le X, Heymach JV, Skoulidis F, et al. Association of EGFR and HER2 exon 20 mutations with distinct patterns of response to immune checkpoint inhibitors in non-small cell lung cancer. J Thorac Oncol. (2018) 36 (15 Suppl):9052. doi: 10.1200/jco.2018.36.15_suppl.9052”. It should be “Negrao MV, Reuben A, Robichaux JP, Le X, Nilsson MB, Wu C, et al. Association of EGFR and HER-2 exon 20 mutations with distinct patterns of response to immune checkpoint inhibitors in non-small cell lung cancer. *J Clin Oncol*. (2018) 36:9052. doi: 10.1200/jco.2018.36.15_suppl.9052”.

The reference for “18” was erroneously written as “Metro G, Baglivo S, Bellezza G, Mandarano M, Gili A, Marchetti G, et al. Efficacy of immune checkpoint inhibitors in patients with advanced non-small-cell lung cancer harboring EGFR exon 20 insertion mutations. Genes (Basel). (2021) 12:679. doi: 10.3390/genes12050679”. It should be “Metro G, Baglivo S, Bellezza G, Mandarano M, Gili A, Marchetti G, et al. Sensitivity to immune checkpoint blockade in advanced non-small cell lung cancer patients with EGFR exon 20 insertion mutations. *Genes (Basel)*. (2021) 12:679. doi: 10.3390/genes12050679”.

The original version of this article has been updated.

